# Realization of a quadrupole topological insulator phase in a gyromagnetic photonic crystal

**DOI:** 10.1093/nsr/nwae121

**Published:** 2024-04-01

**Authors:** Peiheng Zhou, Gui-Geng Liu, Zihao Wang, Shuwei Li, Qindong Xie, Yunpeng Zhang, Subhaskar Mandal, Xiang Xi, Zhen Gao, Longjiang Deng, Baile Zhang

**Affiliations:** National Engineering Research Center of Electromagnetic Radiation Control Materials, Key Laboratory of Multi-spectral Absorbing Materials and Structures of Ministry of Education, University of Electronic Science and Technology of China, Chengdu 611731, China; Division of Physics and Applied Physics, School of Physical and Mathematical Sciences, Nanyang Technological University, Singapore 637371, Singapore; Division of Physics and Applied Physics, School of Physical and Mathematical Sciences, Nanyang Technological University, Singapore 637371, Singapore; National Engineering Research Center of Electromagnetic Radiation Control Materials, Key Laboratory of Multi-spectral Absorbing Materials and Structures of Ministry of Education, University of Electronic Science and Technology of China, Chengdu 611731, China; National Engineering Research Center of Electromagnetic Radiation Control Materials, Key Laboratory of Multi-spectral Absorbing Materials and Structures of Ministry of Education, University of Electronic Science and Technology of China, Chengdu 611731, China; National Engineering Research Center of Electromagnetic Radiation Control Materials, Key Laboratory of Multi-spectral Absorbing Materials and Structures of Ministry of Education, University of Electronic Science and Technology of China, Chengdu 611731, China; Division of Physics and Applied Physics, School of Physical and Mathematical Sciences, Nanyang Technological University, Singapore 637371, Singapore; School of Electrical Engineering and Intelligentization, Dongguan University of Technology, Dongguan 523808, China; Department of Electronic and Electrical Engineering, Southern University of Science and Technology, Shenzhen 518055, China; National Engineering Research Center of Electromagnetic Radiation Control Materials, Key Laboratory of Multi-spectral Absorbing Materials and Structures of Ministry of Education, University of Electronic Science and Technology of China, Chengdu 611731, China; Division of Physics and Applied Physics, School of Physical and Mathematical Sciences, Nanyang Technological University, Singapore 637371, Singapore; Centre for Disruptive Photonic Technologies, The Photonics Institute, Nanyang Technological University, Singapore 639798, Singapore

**Keywords:** quadrupole topological insulator, time-reversal symmetry breaking, corner states, topological phase transition

## Abstract

The field of topological photonics was initiated with the realization of a Chern insulator phase in a gyromagnetic photonic crystal (PhC) with broken time-reversal symmetry (*T*), hosting chiral edge states that are topologically protected propagating modes. Along a separate line of research, a quadrupole topological insulator was the first higher-order topological phase supporting localized corner states, but has been so far limited to *T*-invariant systems, as *T* is a key ingredient in early models. Here we report the realization of a quadrupole topological insulator phase in a gyromagnetic PhC, as a consequence of topological phase transition from the previously demonstrated Chern insulator phase. The phase transition has been demonstrated with microwave measurements, which characterize the evolution from propagating chiral edge states to localized corner states. We also demonstrate the migration of topological boundary states into the continuum, when the gyromagnetic PhC is magnetically tuned. These results extend the quadrupole topological insulator phase into *T*-broken systems, and integrate topologically protected propagating and localized modes in a magnetically tunable photonic crystal platform.

## Teaser

Realization of a quadrupole topological insulator (QTI) phase in a gyromagnetic photonic crystal broadens the realm of QTI phases to encompass time-reversal symmetry-broken systems and holds the potential to integrate topologically protected unidirectional propagating and localized states in a single photonic platform.

## INTRODUCTION

Topological photonics is a subfield of photonics dedicated to exploring the topological states of light [1–4]. This field was initiated more than 10 years ago with a gyromagnetic photonic crystal (PhC) that exhibited the Chern insulator (CI) phase with broken time-reversal symmetry (*T*) [5–8]. When biased by external magnetic fields, the gyromagnetic PhC opens a topological band gap characterized by the non-zero Chern number, hosting chiral edge states that are unidirectionally propagating modes along the 1D edges of a sample [5–10]. Such photonic chiral edge states exhibit robustness against defects and disorder, being technologically promising in various applications such as topological lasers [11–13] and photonic circuits [8,14].

Higher-order band topology is a recent breakthrough in topological physics [15]. This line of research began with the famous Benalcazar-Bernevig-Hughes (BBH) model that exhibits a quantized quadrupole moment as the bulk topology [16,17]. Such an insulator is called a quadrupole topological insulator (QTI), with its topological states localized at its 0D corners [16–24]. The QTI phase has been realized in a variety of platforms, such as microwave circuits [20], mechanical metamaterials [23], electric circuits [18], acoustic crystals [21,24] and photonic lattices [19,22]. So far, all previous QTI realizations have been limited to *T*-invariant systems [18–24], since *T* is a key ingredient for the quadrupole moment there. As a result, a *T*-broken QTI has never been realized, and it remains unclear what *T* breaking can be used for in such a *T*-broken QTI phase.

Recent theories have shown that the quadrupole topology can potentially occur in a *T*-broken system such as a gyromagnetic PhC [25,26]. A gyromagnetic PhC with broken *T* offers the advantage of eliminating negative coupling, which is a challenge for a PhC but is an essential component in previous realizations. Moreover, this gyromagnetic PhC has the potential to realize topological phase transition between CI and QTI, which in turn permits the integration of topologically protected unidirectional propagating and localized states in a single photonic platform. Note that there are also a variety of higher-order localized states that arise from a non-zero dipole moment in PhCs [27–30]. However, such states belong to higher-order topological phases ultimately distinct from QTI, as deliberately classified in a recent theoretical work [30].

Notably, the occurrence of such a phase transition between CI and QTI also provides insights into 3D higher-order Weyl semimetals, particularly when treating these 3D semimetals as effectively 2D systems governed by specific *k_z_* values [31–33]. However, it is worth noting that the direct experimental validation of such a phase transition requires *T* breaking, a feat that has yet to be achieved.

Here, we present the experimental realization of a QTI in a gyromagnetic PhC. Compared to previous QTI realizations that are all *T* invariant, we emphasize the uniqueness of our work—the intentional *T* breaking through the application of external magnetic fields. Firstly, our work serves as the first QTI realization in a *T*-broken system. Unlike QTI models governed by *T* invariance, such as the BBH model, our experimental realization broadens the realm of QTI phases to encompass *T*-broken systems. Secondly, the broken *T* allows us to demonstrate the topological phase transition between QTI and CI phases. This transition holds the potential to integrate topologically protected unidirectional propagating and localized states in a single photonic platform. Finally, we further manipulate *T* breaking as a unique degree of freedom to manipulate topological boundary states. By tuning magnetic fields, we show that topological corner states can migrate into bulk bands and remain localized. This approach contrasts with previous demonstrations of migrating/constructing topological boundary states into/in the bulk, which were based on *T*-invariant approaches [34–37].

## RESULTS AND DISCUSSION

**Figure 1.**
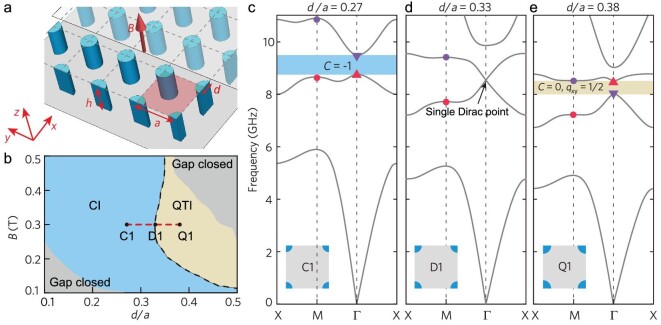
Design of a gyromagnetic photonic crystal with topological phase transition from Chern insulator (CI) phase to quadrupole topological insulator (QTI) phase. (a) A photonic square lattice consisting of gyromagnetic quarter-cylinders with diameter *d* and height *h* = 8 mm. The lattice constant is *a* = 14.3 mm. An external magnetic field *B* is applied along + *z* direction. The lattice is placed between two metal planes (in gray) with the upper plane half-covered and transparent for visualization. (b) Phase diagram. The topological phase transition occurs along the dashed black line. Black dots indicate three distinct phases at *B* = 0.3 T: C1 is a CI phase; D1 denotes the phase transition point; Q1 has QTI phase. The photonic bandgap also closes at the gray regions. (c–e) Band structure evolution induced by *d/a* at *B* = 0.3 T. Colored dots and triangles in the band structure diagrams indicate the band inversion of second and third eigenstates at Γ points. Insets are the unit cell configurations of each crystal.

The designed gyromagnetic PhC is depicted in Fig. 1a. The unit cell consists of four quarters of a gyromagnetic ferrite cylinder with diameter *d*, each of which is located at a corner of a square with side *a*. A uniform magnetic field *B* is applied along the *z* direction to break the *T*. The entire PhC is sandwiched between two parallel metal planes, and the electric fields are polarized along the *z* direction.

As *B* and *d* vary, both the CI phase and the QTI phase can emerge in the gyromagnetic PhC, as shown in the phase diagram plotted in Fig. 1b. Here we fix *B* = 0.3 T, and plot the band dispersions of the PhC at *d/a* = 0.27 (C1), 0.33 (D1) and 0.38 (Q1) in Fig. 1c–e, respectively. The calculated gap Chern number (*C*) and quadrupole moment (*q_xy_*) have been indicated in the same figure (see Section S1 for their calculation).

As shown in Fig. 1c for the C1 state, a complete band gap occurs between the second and the third bands, and its corresponding non-zero gap Chern number *C* = −1 manifests C1 as a CI phase. By increasing *d* to reach the D1 state (Fig. 1d), the gap closes and forms a single Dirac point at the center of the Brillouin zone (Γ point) [38] (see Section S2 for its verification), which can be effectively treated as a magneto-optical near-zero index medium [39]. Further increasing *d* to reach Q1 (Fig. 1e), a complete gap reappears between the second and third bands, featuring a trivial Chern number yet a non-trivial quadrupole moment *q_xy_* = 1/2, indicating that Q1 corresponds to a QTI phase. During the phase transition process between the CI phase (C1) and the QTI phase (Q1), a band inversion occurs between the second and third bands, evidenced by exchanged eigenstates at the Γ point (marked as purple and red triangles in Fig. 1c and e; see Section S2 for the profiles of eigenstates). In contrast, previously discussed trivial-nontrivial QTI phase transition relies on the band inversion at the M point [25]. The CI-QTI phase transition can also be induced by tuning the external magnetic field strength *B* (see Section S2 for the phase transition).

**Figure 2.**
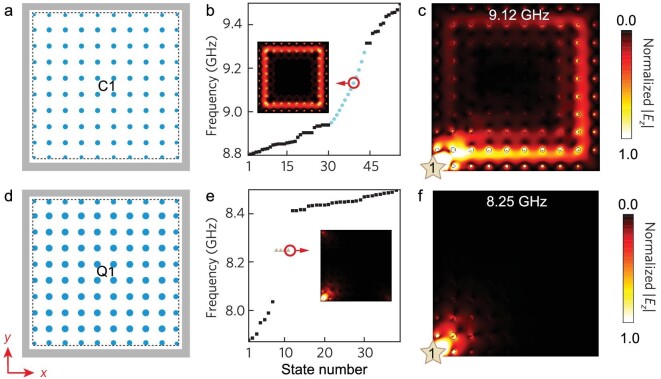
Simulation of in-gap boundary states. (a)/(d) Square lattice for C1/Q1. The samples with 9 × 9 unit cells are enclosed by PECs in simulation. Here the PEC edges are placed away from the lattice edges (dotted lines) with a constant distance of 0.21*a*. (b)/(e) Numerically simulated eigenstates of the C1/Q1 sample. Edge states (circles in (b)) and four corner states (triangles in (e)) are found in the bandgap spectrum with one typical profile shown in the insets. Other bulk states are colored in black. (c)/(f) Simulated *E_z_* field distributions for corner excitation of the C1/Q1 sample. Colored stars denote the source antenna (labeled ‘1’).

Next, we study the boundary modes of C1 and Q1 by constructing two finite samples, as plotted in Fig. 2a and d, respectively. Here, semi- and quarter-cylinders near the boundaries of the samples are replaced by full circular cylinders with preserved areas of cross sections to facilitate the experimental fabrication. Note that simulation results have shown that such shape deformations have no essential influence on the eigenmodes and experimental observations (see Section S3 for more details). Perfect electric conductors (PECs) are introduced to all boundaries to prevent wave leakage into the surrounding environment. Figure 2b shows the eigenmodes solved for the C1 sample in Fig. 2a. Clearly, the gap of the bulk states has been populated with chiral edge states, indicating that the C1 sample belongs to the CI phase. With a source antenna placed near a corner of the C1 sample (labeled ‘1’ in Fig. 2c), chiral edge states are found propagating counterclockwise along the boundaries with negligible reflection; the decay of the propagating edge states is the result of unavoidable material losses (see Section S3 for more details). For the Q1 sample, Fig. 2e shows that only four degenerate corner modes exhibit in the bulk gap, indicating the emergence of a QTI phase. The corner excitation in Fig. 2f thus results in highly localized corner states.

**Figure 3.**
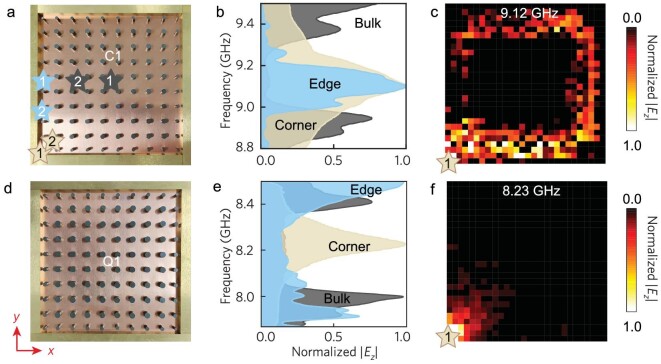
Observation of in-gap boundary states. (a)/(d) Photograph of a square sample for C1/Q1. The PhC samples are enclosed by copper bars with the same geometrical set-ups indicated in Fig. 2a/d. Colored stars denote the source antenna (labeled ‘1’) and probe antenna (labeled ‘2’) in three measurements for corner, edge and bulk transmissions. (b)/(e) Measured frequency-resolved transmissions for the C1/Q1 sample. (c)/(f) Measured field distributions excited by port ‘1’ for the C1/Q1 sample.

Experimentally, the yttrium iron garnet ferrite cylinders are employed to construct the C1/Q1 sample in Fig. 3a/d. The samples are covered with copper claddings that function as PECs. We measure the transmissions of the bulk, edge and corner states by placing a pair of antennas, as illustrated by labels ‘1’ and ‘2’ in Fig. 3a, into the fabricated gyromagnetic PhCs. As shown in Fig. 3b, the measured edge transmission for the C1 sample dominates at around 8.96 GHz–9.27 GHz, consistent with the eigensolver predictions in Fig. 2b. We also put a source antenna near a corner of the sample and scanned the excited field distribution. When the oscillating frequency (9.12 GHz) lies in the bulk gap, the measured electric fields in Fig. 3c propagate robustly along the counterclockwise direction, in agreement with the simulation results in Fig. 2c. There is also the decay of the propagating edge states resulting from unavoidable material losses.

For the measured transmissions in Fig. 3e for the Q1 sample, both the bulk and edge transmissions exhibit obvious dips around 8.04 GHz–8.38 GHz, indicating the absence of the edge states in the bulk gap. However, a significant peak of the corner transmission appears at 8.23 GHz, manifesting the emergence of corner states. Note that a frequency shift of 0.02 GHz from the simulation results in Fig. 2e, which may be caused by fabrication errors and experimental imperfections, is generally negligible. Further field scanning results have visualized the strongly localized corner states directly, as shown in Fig. 3f. These results have experimentally corroborated the topological phase transition between a CI phase with chiral edge states and a QTI phase with localized corner states in a PhC platform. With further proper choice of CI and QTI parameters, integrating chiral edge states and corner states in the same platform can be accomplished by creating coupling paths in CI-QTI heterostructures (see Section S4 for the integration of topological states).

**Figure 4.**
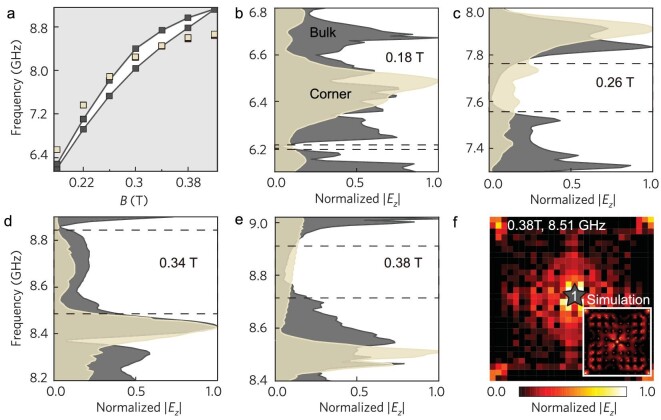
Observation of corner states in the bulk continuum. (a) Numerically simulated eigenstates of the QTI sample with *d/a* = 0.38. The gray/white region denotes the bulk continuum/bandgap. Tan squares represent corner states. (b–e) Measured bulk and corner transmission spectra at 0.18 T, 0.26 T, 0.34 T and 0.38 T, respectively. Note that the corresponding results for the QTI sample at 0.3 T are shown in Fig. 3e. (f) Measured field distributions excited by port ‘1’ at 0.38 T and 8.51 GHz. Inset is the corresponding simulation result. The colored star denotes the source antenna (labeled ‘1’).

Corner states embedded in the continuum [34–37], which is a distinctive characteristic facilitated by the higher-order topology, can also been realized in our platform. In previous proposals of *T*-invariant higher-order topological phases, the shift of corner states can be realized by modifying material and structural parameters [40,41]. In our platform, the eigenfrequencies of the corner states can be shifted simply by tuning *B*. Here, we fix the PhC with *d/a* = 0.38 and vary *B* from 0.18 T to 0.42 T. As shown in Fig. 4a, the band gap separates the bulk continuum into the lower and higher frequency ranges. Increasing *B* continuously shifts the corner states from the higher frequency range into the gap, and then into the lower frequency range. This property has been experimentally verified by measuring the bulk and edge transmissions for *B* = 0.18 T, 0.26 T, 0.34 T and 0.38 T, as shown in Fig. 4b–e, respectively. Specifically, for *B* = 0.38 T in Fig. 4e, the peak of the corner transmission emerges at 8.51 GHz, which is in the lower range of the bulk states. Consequently, a source antenna at 8.51 GHz will simultaneously excite both the bulk and corner states in a finite sample, as verified by the measured and simulated field distributions in Fig. 4f (see Section S6 for more details on the *Q* factor of the embedded corner states).

We have thus realized a QTI phase by a gyromagnetic PhC and revealed its topological phase transition from the CI phase. Both the in-gap and bulk-embedded corner states have been observed. These results extend the quadrupole topology to *T*-broken systems. The integration of chiral edge states and corner states in the PhC platform provides an opportunity to switch between topologically protected propagating and localized photonic modes.

## METHODS

The gyromagnetic PhC cylinders are fabricated by low loss commercial yttrium iron garnet (YIG) ferrite material with a microwave relative permittivity of 14.3–0.0028 i. Magnetic measurements show that the saturation magnetization is *M*_s_ = 1780 Gauss and the gyromagnetic resonance loss width is 35 Oe. In the bulk, the cylinders have a radius of 2.72 mm for Q1 (1.93 mm for C1). At four boundaries, the edge cylinders have a radius of 1.92 mm for Q1 (1.36 mm for C1), and the corner cylinders have a radius of 1.36 mm for Q1 (0.96 mm for C1). During measurements, the samples are uniformly biased by a static magnetic field applied perpendicular to the cylinders.

In numerical studies, the band structure, bulk/edge/corner transmissions, eigenstate dispersion and field distributions are obtained by commercial software package COMSOL Multiphysics. For the sake of simplicity, the frequency dispersion and material losses are neglected in band structure calculations (see Section S3 for more details).

## Supplementary Material

nwae121_Supplemental_File
