# Supplementary material for: Realization of a quadrupole topological insulator phase in a gyromagnetic photonic crystal
Source: Natl Sci Rev. 2024 Apr 1;11(11):nwae121. doi: 10.1093/nsr/nwae121 (PMC11493087; doi:10.1093/nsr/nwae121)
Supplement: nwae121_Supplemental_File [file nwae121_supplemental_file.docx]

***Supplementary Material***

**Realization of a quadrupole topological insulator phase in a gyromagnetic photonic crystal**

Peiheng Zhou^1^, Gui-Geng Liu^2,†^, Zihao Wang^2^, Shuwei Li^1^, Qindong Xie^1^, Yunpeng Zhang^1^, Subhaskar Mandal^2^, Xiang Xi^3^, Zhen Gao^4^, Longjiang Deng^1,†^, and Baile Zhang^2,5,†^

^1^National Engineering Research Center of Electromagnetic Radiation Control Materials, Key Laboratory of Multi-spectral Absorbing Materials and Structures of Ministry of Education, University of Electronic Science and Technology of China, Chengdu 611731, China

^2^Division of Physics and Applied Physics, School of Physical and Mathematical Sciences, Nanyang Technological University, 21 Nanyang Link, Singapore 637371, Singapore

^3^School of Electrical Engineering and Intelligentization, Dongguan University of Technology, Dongguan, 523808, China

^4^Department of Electronic and Electrical Engineering, Southern University of Science and Technology, Shenzhen 518055, China.

^5^Centre for Disruptive Photonic Technologies, The Photonics Institute, Nanyang Technological University, 50 Nanyang Avenue, Singapore 639798, Singapore

^†^ Corresponding authors. Emails: guigeng001@e.ntu.edu.sg; denglj@uestc.edu.cn; blzhang@ntu.edu.sg

**1. Calculation of topological invariants**

For Chern insulators (CIs), we first calculate the Chern number of the first and second bands for photonic crystals (PhCs) in the blue part of Fig. 1(b) in the main text. The Chern number is calculated by integrating the Berry curvatures over the first Brillouin Zone of our 2D PhCs. The Berry curvature is defined as $\Omega_{n}\left(\mathbf{k}\right)=\nabla_{\mathbf{k}}\times F_{n}\left(\mathbf{k}\right)$, where $F_{n}\left(\mathbf{k}\right)=i\langle u_{n,\mathbf{k}}|\nabla_{\mathbf{k}}|u_{n,\mathbf{k}}\rangle$ is the Berry connection and $u_{n\text{，}\mathrm{k}}$ is the Bloch state for the *n*-th band. The Chern number is given by $\frac{1}{2\pi}\iint dk_{x}dk_{y}\Omega_{n}\left(\mathbf{k}\right)$. Correspondingly, the first band has Chern number of 0, and the second band has a nontrivial value of -1. The gap Chern number of the photonic bandgap between the second and third bands is thus obtained by adding these two values together, i.e. *C* = 0 + (-1) = -1.


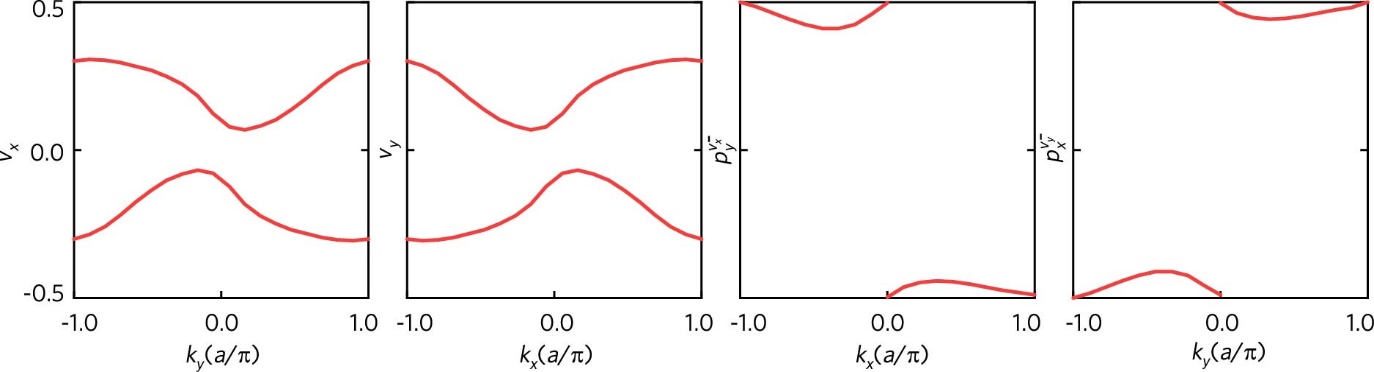


FIG. S1. The (nested) Wannier bands *v_x, y_* ($p_{y,x}^{v_{x,y}^{-}}$) for Q1. The first and second bands are calculated. Here, the nested Wannier bands are calculated for the $v_{x}^{-}$and $v_{y}^{-}$sectors.

For quadrupole topological insulators (QTIs), we demonstrate the nontrivial band topology using the (nested) Wannier bands in Wilson loop approach [1]. We consider the Wilson loop operators in the *x* direction. After calculating the Berry connection differently by $\left[F_{\mathbf{k}}^{x}\right]^{m,n}=\langle u_{m,\mathbf{k}+\Delta k_{x}}|u_{n,\mathbf{k}}\rangle$, with Δ*k_x_*= 2π/*n_k_* being the difference of the discretized momentum and *m*, *n* denoting the bands considered, the Wilson loop is obtained by $W_{\mathbf{k}}^{x}=\prod_{i=0}^{n_{k}-1}F_{\mathbf{k}+i\Delta k_{x}}^{x}$. Then, the Wannier bands are calculated by $W_{\mathbf{k}}^{x}|v_{x,\mathbf{k}}^{j}\rangle=e^{2\pi iv_{x}^{j}\left(k_{y}\right)}|v_{x,\mathbf{k}}^{j}\rangle$ , with *j* the band index, and $|v_{x,\mathbf{k}}^{j}\rangle$the eigenvector of the Wilson loop. The *y* component $v_{y,\mathbf{k}}^{j}$ can be obtained similarly. As the Wannier bands are gapped, the nested Wannier bands can be calculated in two sectors: $|w_{x,\mathbf{k}}^{\pm}\rangle=\sum_{n} |u_{n,\mathbf{k}}\rangle\left[v_{x,\mathbf{k}}^{\pm}\right]^{n}$, with ± indicating the Wannier sectors and $\left[v_{x,\mathbf{k}}^{\pm}\right]^{n}$the *n*th component of the Wannier eigenvector$|v_{x,\mathbf{k}}^{\pm}\rangle$. For sector ‘-’, we define $\left[F_{y,\mathbf{r}}^{-}\right]^{mn}=\langle w_{x,\mathbf{k}+\Delta k_{y}}^{-,m}|w_{x,\mathbf{k}}^{-,n}\rangle$ where Δ*k_y_*= 2π/*n_k_*, and *m*, *n* = 1…*N_w_* indicate the bands within this sector. The nested Wilson loops along the *k_y_* direction are defined by $\tilde{W}_{y,\mathbf{k}}^{-}=\prod_{i=0}^{n_{k}-1} F_{y,\mathbf{k}+i\Delta k_{y}}^{-}$. To find the phase of the nested Wilson loop eigenvalues $e^{2\pi ip_{y}^{v_{x(k_{x})}^{j}}}$, we diagonalize the nested Wilson loop operator. Finally, the nested Wannier band polarization is obtained by $p_{y}^{v_{_{x}}^{-}}\left(k_{x}\right)=\sum_{j=1}^{N_{w}} p_{y}^{v_{x(k_{x})}^{j}}=\frac{-i}{2\pi}\mathrm{lndet}\tilde{W}_{y,\mathbf{k}}^{-}$. Similar process can be applied to find $p_{x}^{v_{_{y}}^{-}}\left(k_{y}\right)$. Taking Q1 for example, the calculated results are shown in Fig. S1. It is clear that the Wannier bands are gapped, and their polarization within one sector are quantized to be 1/2. Therefore, we obtain bulk quadrupole moment $q_{xy}=2p_{x}^{v_{y}^{-}}p_{y}^{v_{x}^{-}}=1/2$.

**2. Phase transition**

**2.1 Berry curvature verification of the single Dirac point**

Following the previous definition of Berry curvature, we further calculate it for our gyromagnetic PhCs around the phase transition point (D1 of *d / a* = 0.33, *B* = 0.3 T) as well as the C1 and Q1 phases. It is clear that the Berry curvatures of the second band have non-zero values around Γ point in Fig. S2. For CIs, the integral of Ω gives -2π, and thus we find *C* = -1. Increasing *d / a*, the distribution of the Berry curvature shrinks to Γ point, and then flips its sign after phase transition at *d / a* = 0.33. Afterward, the positive Ω around Γ is balanced by negative Ω away from the center to give *C* = 0. Therefore, the critical point D1 must be a single Dirac point with no pairs in the Brillouin zone.


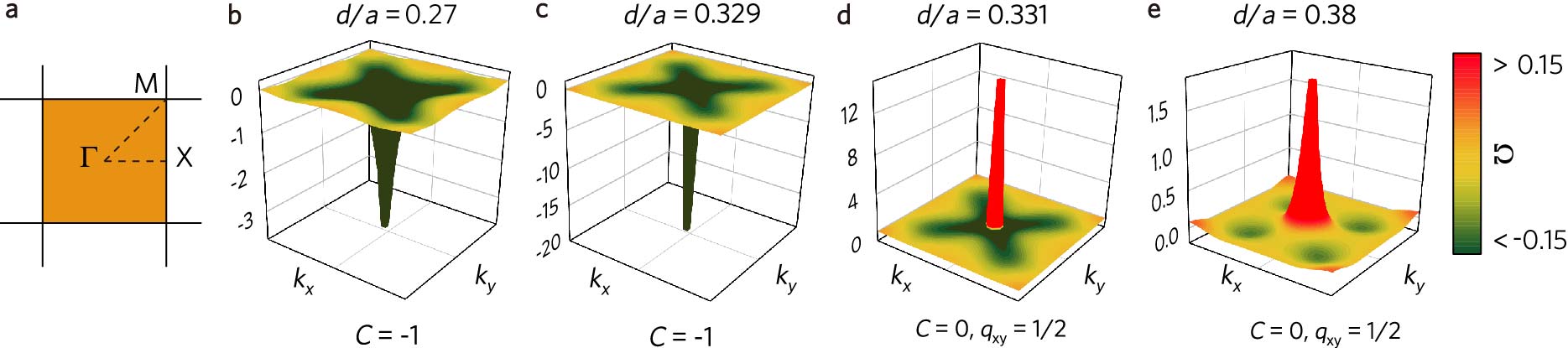


FIG. S2. Berry curvature of the second band in momentum space. (a) Brillouin zone of the square lattice of our gyromagnetic photonic crystals (PhCs). (b)-(e) Numerical Berry curvature for PhCs with *d / a* = 0.27 (C1), 0.329, 0.331, and 0.38 (Q1). The Berry curvature results are calculated in the Brillouin zone shown in (a), where -π/*a* ≤ *k_x_*, *k_y_* ≤ π/*a*. For all the samples, *B* = 0.3T.

**2.2 Eigenstate phase profiles, band inversion, and magnetic field induced phase transition**


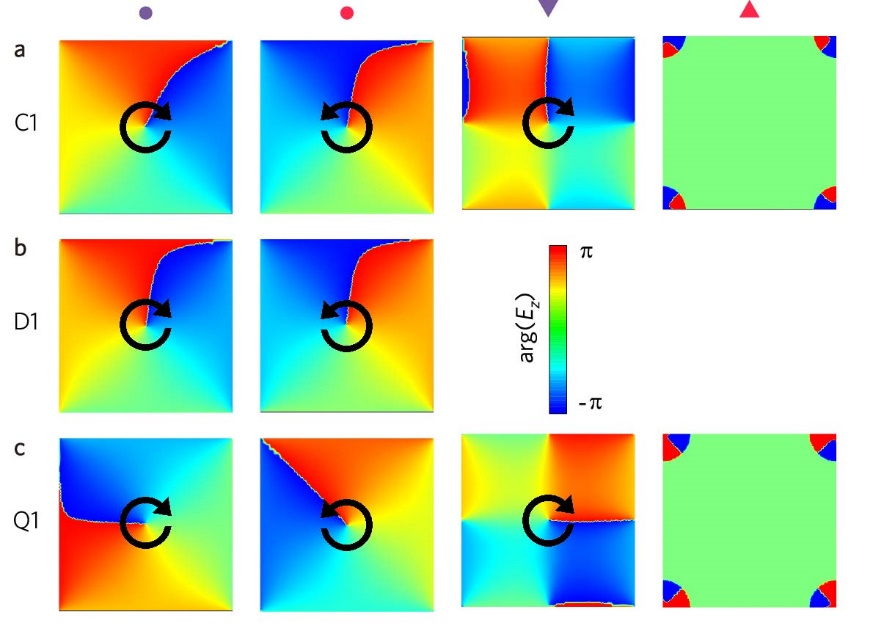


FIG. S3. Verification of the band inversion by eigen states. (a) / (b) / (c) Phase profiles for the second band and third band eigenstates at M (dots) and Γ (triangles) points as marked in Fig. 1(c-e) in the main text for C1 (CI phase) / D1 (single Dirac point) / Q1 (QTI phase).

As indicated in Figs. 1(c-e) in the main text, the CI-QTI phase transition for *B* = 0.3 T is characterized by the band inversion at Γ point, i.e. the switch of eigenstates. Meanwhile, the second band and third band eigenstates at M point do not switch. To verify these conclusions, we plot the phase profile of the second band and third band eigenstates at M and Γ points for C1, D1, and Q1 topological phases in Fig. S3. At M point, the phase profiles of the second band for all three phases show a winding of +2π, so they are clarified in one category (labeled as red). Similarly, the phase profiles of the third band show a winding of -2π, so they are identified as another category (labeled as purple). At Γ point, we define the two categories of the phase profiles for C1 and Q1: the one with a winding of -2π (labeled as purple) and the other one with no winding (labeled as red).

At a fixed *d* / *a*, the CI-QTI phase transition can also be induced by varying *B*. For example, at *d* / *a* = 0.34, topological phase transition of our gyromagnetic PhCs occurs at two gap closing points, i.e. *B* = 0.23 T and 0.37 T in Fig. S4. Band inversion at Γ point features the emergence of quadrupole topology for *B* = 0.3 T. Following the calculation of topological invariant, we find that bulk quadrupole moment *q_xy_* = 1/2. Quadrupole topological phase is thus characterized. After phase transition caused by either increasing or decreasing *B*, CIs are achieved with a gap Chern number of *C* = -1, consistent with Fig. 1(b) in the main text. We thus show that varying *B* is another degree of freedom in controlling quadrupole moments.


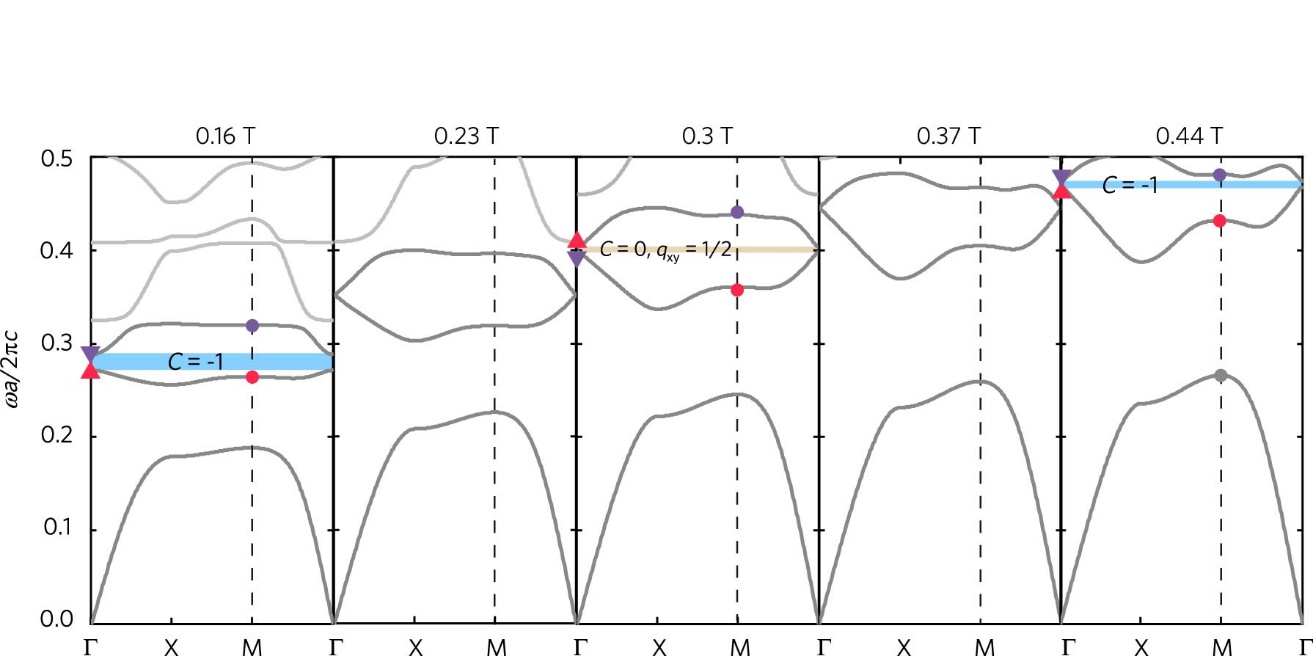


FIG. S4. Phase transition induced by *B* varying. Photonic band structure of gyromagnetic photonic crystals with *B* changing from 0.16T to 0.44T, when *d* / *a* = 0.34. The blue and tan regions indicate the nontrivial CI and QTI band gaps, respectively. Colored dots and triangles in the band structure diagrams represent the eigenstates with same features indicated in Fig. S3.

**3.** **Materials and experimental set-ups**

The yttrium iron garnet (YIG) ferrite cylinders have a relative permittivity of 14.3 and a dielectric loss tangent of 0.0002. With the saturation magnetization *M*_s_ = 1780 Gauss, and the gyromagnetic resonance loss width 35 Oe, the relative magnetic permeability of the YIG cylinders biased by an external magnetic field along the *z*-axis has the form

$\overleftrightarrow{\mu}=\left[\begin{matrix} \begin{matrix} \mu_{r} \\ -i\kappa\\ 0 \end{matrix} & \begin{matrix} i\kappa\\ \mu_{r} \\ 0 \end{matrix} & \begin{matrix} 0 \\ 0 \\ 1 \end{matrix} \end{matrix}\right]$,

where$\mu_{r}=1+\frac{\left(\omega_{0}+i\alpha\omega\right)\omega_{m}}{\left(\omega_{0}+i\alpha\omega\right)^{2}-\omega^{2}}$, $\kappa=\frac{\omega\omega_{m}}{\left(\omega_{0}+i\alpha\omega\right)^{2}-\omega^{2}}$, $\omega_{m}=\gamma\mu_{0}M_{s}$, $\omega_{0}=\gamma\mu_{0}H_{z}$, $\mu_{0}H_{z}$= *B* is the external magnetic flux density along the *z*-axis, *γ* = 1.76 × 10^11^ s^-1^T^-1^ is the gyromagnetic ratio, *α* = 0.0088 is the damping coefficient, and *ω* is the operating frequency. Accordingly, there exists moderate magnetic loss that causes decay in propagating waves but does not affect the underlaying physics. For example, Fig. S5 shows the dispersion of magnetic permeability within the operational frequency range of sample C1 (*B* = 0.3 T). At the frequency demonstrating chiral edge states, i.e. 9.12 GHz in Figs. 2(c) and 3(c) in the main text, the loss tangent is 0.167 and 0.106 for *μ_r_* and *κ* respectively. The loss tangent is moderate. However, after propagating over four edges each of length 9.42*a* (*a* = 14.3 mm), the attenuation of wave energy is observable in both simulation and experiments.

In the simulation, the phase transition diagram, bulk / edge / corner transmissions, eigenstate dispersion and field distributions (simulation results in Fig. 1(b), Fig. 2, and Fig. 4 in the main text; Figure S7 in the Supplementary Material) are calculated using the finite element software COMSOL Multiphysics with above material parameters. For band structure calculation (simulation results in Figs. 1(c-e) in the main text and Figs. S1-S4 in the Supplementary Material), we mainly focus on the feature of band gap region, so the material parameters, i.e. *μ*_r_ and *κ*, are treated as non-dispersion quantities with values chosen at the center frequency of the band gap.


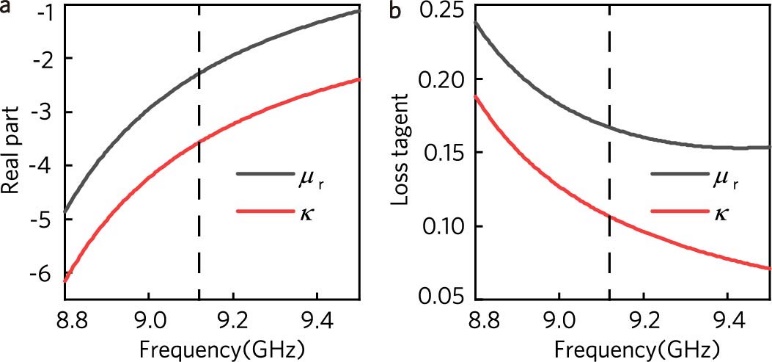


FIG. S5. Dispersion of magnetic permeability for YIG material. (a) Real part of the diagonal component (*μ_r_*) and off-diagonal component (*κ*) of the magnetic permeability. (b) Loss tangent of the diagonal component (*μ_r_*) and off-diagonal component (*κ*) of the magnetic permeability. The dotted lines indicate the frequency of 9.12 GHz.


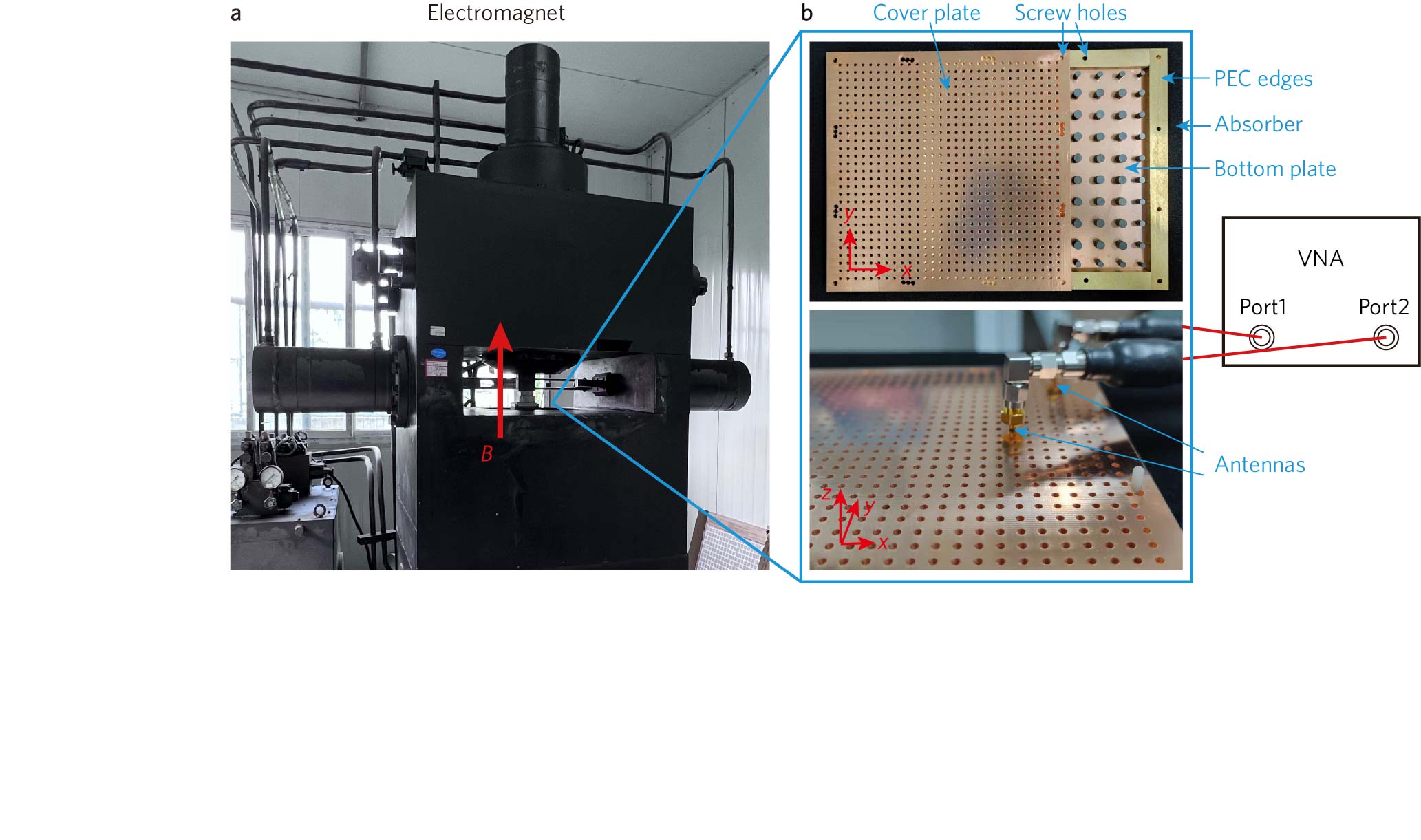


FIG. S6. Experimental setups. (a) Photograph of the electromagnet that provide large-area, stable, and variable magnetic field. (b) Field-mapping experimental setups. Upper panel in the blue rectangle frame is the picture of our QTI sample with cover plate half-shifted to visualize the inner structure. Bottom panel indicates how the source antenna and probe antenna insert into the sample. Both antennas are connected to a Vector Network Analyzer. Screw holes drilled through the cover plate and PEC edges are used to fix the whole structure.

In the experiment, the measurement setup is the same as that used in the supplementary reference [2,3]. We use a double-tuned air gap alterable electromagnet, with the magnetic field in the vertical direction and pole diameter 250 mm (see Fig. S6(a)). Thus, our square samples of side length (9+0.21×2)*a* ≈ 134.71 mm (*a* = 14.3 mm is the lattice constant) can be safely magnetized on the pole surface. The electromagnet contains water-cooling structure, which stabilizes the magnetic field for a long time and provides reliable magnetic environment for microwave measurements. All experiments are conducted in parallel plate waveguides wrapped by perfect electrical conductor (PEC) edges (see Fig. S6(b) as an example). Copper bars form the PEC edges. Within the waveguides, gyromagnetic cylinders are fixed to the bottom copper plate. Pixeled holes with a diameter of 2 mm and a period of 5 mm are drilled through the cover copper plate. Such holes serve as antenna ports in the microwave measurements. Note that the holes are at deep subwavelength scale, that they have negligible effect on the electromagnetic-wave mode inside the parallel waveguide [2,3]. This design provides great flexibility for placing the source antenna and probe antenna at different positions within the area of the sample without moving the waveguide assembly. During measurement, the whole structure is embedded in microwave foam absorber to eliminate the effect of leaky wave.


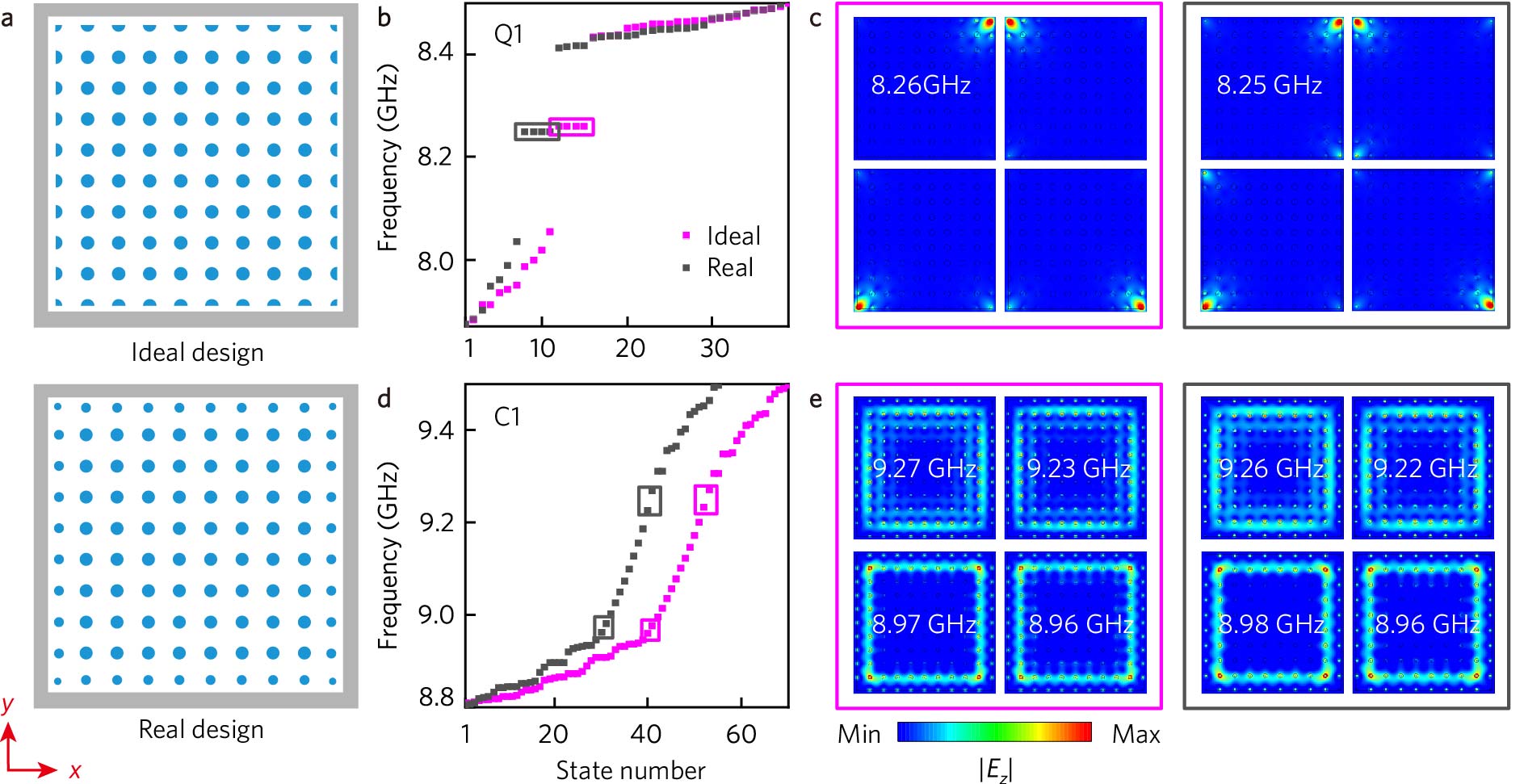


FIG. S7. Consistency between the ideal design and the real design of gyromagnetic PhC samples. (a) Configuration of the ideal design and the real design. The square samples with 9 × 9 unit cells are enclosed by PECs with a constant distance 0.42*a*. Here the PEC edges are colored grey in the panel. Quarter- and semi-cylinders along the lattice edges in the ideal design are replaced by cylinders of preserved area of cross sections in realization. (b-c) / (d-e) Numerically simulated eigenstates of the Q1 / C1 sample in ideal and real designs. The *E_z_* field profile of four degenerate corner states in the ideal design (in cyan frame, left panel of (c)) / real design (in black frame, right panel in (c)) are obtained at 8.26 GHz / 8.25 GHz for Q1. For C1 in (e), four edge states close to the band gap are chosen to display their field profiles from 8.96 GHz to 9.27 GHz (8.96 GHz to 9.26 GHz) in the ideal design (real design).

To facilitate fabrication, we have replaced the ideal design (containing quarter- and semi- cylinders) with a more practical design (with full circular cylinders). It is important that the ideal design is equivalent to the real design in terms of topological features. Here we show the equivalency by eigenstates comparison in Fig. S7. For the two fundamental topological phases in our PhC, i.e. QTI and CI, we choose two samples: Q1 with *d* / *a* = 0.38, *B* = 0.3 T and C1 with *d* / *a* = 0.27, *B* = 0.3 T, the same as those in the main text. Clearly, the two designs have consistent corner states in the bandgap with only a negligible frequency shift of 0.01 GHz for Q1 in Figs. S7(b-c). The perturbations in edge and corner elements are so small that the electromagnetic waves can hardly distinguish them. For C1, the edge states chosen in Fig. S7(d) are the ones closest to the upper and lower boundaries of the band gap. Their positions in the spectrum identify the consistency of band gaps. Furthermore, the two designs show almost identical results in edge states profile in Fig. S7(e). Hence, we can conclude that the real design can safely replace the ideal design without scarifying key topological features.

**4. Integration of topological states**


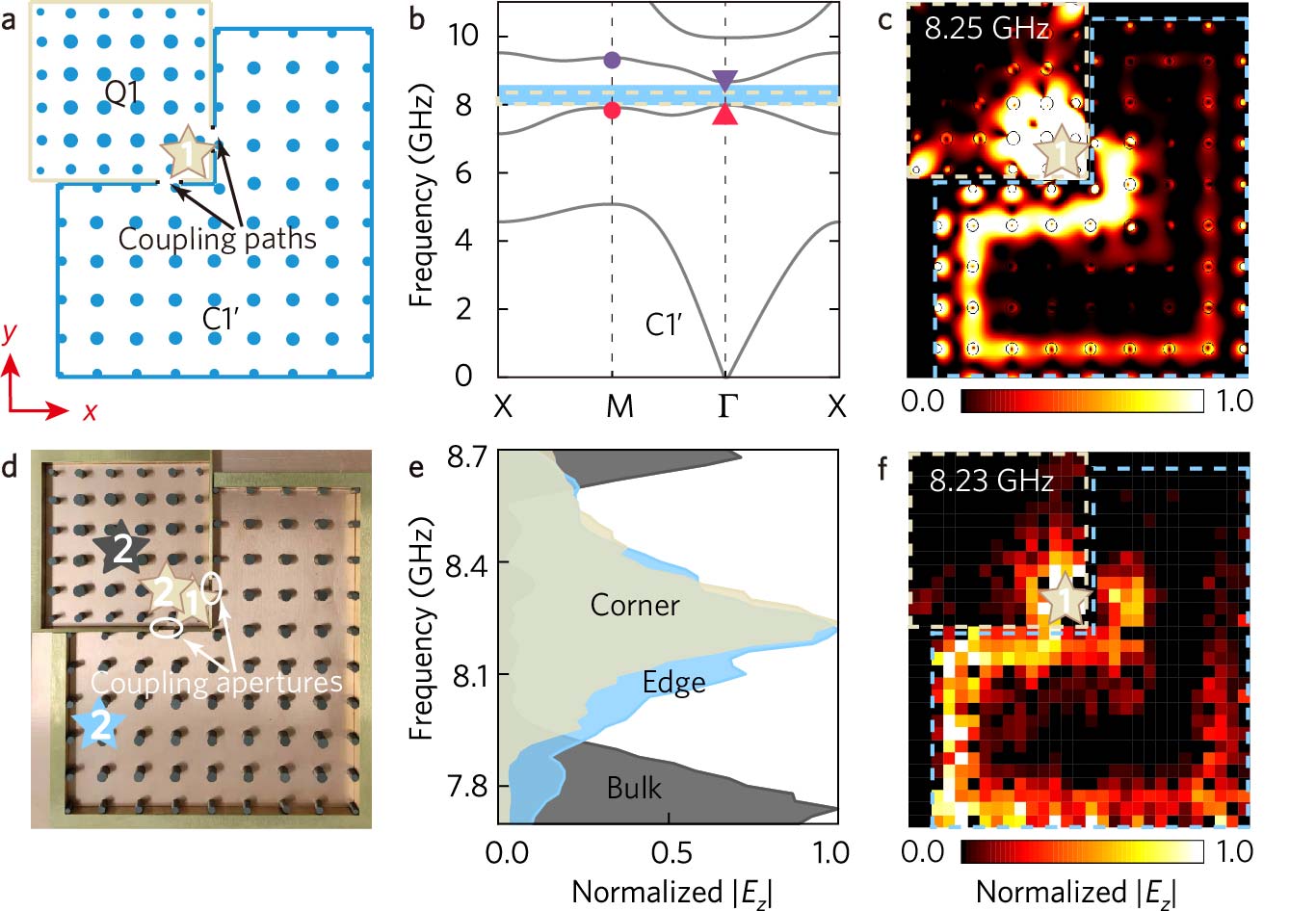


FIG. S8. Coupling of topological states. (a) Schematic of a heterostructure composed of a QTI (Q1) and a CI (C1’). Tan / blue lines indicate the PEC edges that wrap the Q1 / C1’ square lattice. Two lattices are connected to each other through two coupling paths. C1’ has a lattice constant *a*’ = 16.8 mm, *d* / *a*’ = 0.26, *h* = 8 mm, *B* = 0.3T, and zero distance between lattice edges and PEC edges. (b) Band structure of C1’. A non-trivial photonic bandgap (blue region) full covers that of Q1 (region framed by tan dotted line). (c) Simulated *E_z_* field distributions for corner excitation. The tan / blue dotted lines frame Q1 / C1 region. (d) Photograph of the Q1-C1’ heterostructure sample. (e) / (f) Measured frequency-resolved transmissions / field distribution for Q1-C1’ sample. Colored stars denote the source antenna (labelled as “1”) and probe antenna (labelled as “2”) in three measurements for corner, edge, and bulk transmissions. Note that the source antenna is fixed at the corner for all the measurements.

With proper choice of CI and QTI, it is doable to have chiral edge states and corner states in the same frequencies. Then, they can be coupled to each other by creating coupling paths in CI-QTI heterostructures. For example, a Q1-C1’ heterostructure is studied in Fig. S8. First, the lattice parameters of C1 in the main text are optimized to create sample C1’, that hosts chiral edge states in the photonic bandgap of Q1. The bulk band structure in Fig. S8(b) clearly shows such a frequency region of 8.00 GHz - 8.65 GHz. Then, the Q1 region is connected to C1’ region by two coupling paths near one corner (see Fig. S8(a), the opening width of these paths is 0.7*a*). Consequently, a point source located near this corner excites corner states in Q1 region at 8.23GHz and couples to the C1’ region. In Fig. S8(c), we observe numerically the counter-clockwise propagating chiral edge states generated by such coupled corner states. Experimentally, the coupling paths are realized by apertures (0.7*a* × 0.8*h* × 0.1*a*, Fig. S8(d)) drilled on the copper slots that work as PEC edges. Microwave transmission measurements (see Fig. S8(e)) provide strong evidences to the bulk bandgap and middle-gap corner states in Q1, as well as the edge states in C1’. Field mapping results in Fig. S8(f) demonstrate directly the integration of chiral edge states and corner states in this structure.

**5. Comparison between corner / defect states with disorders**

To further prove the topological protection of the corner states, we introduce disorder to our QTI models by adjusting the radii r of gyromagnetic semi- cylinders 1 and 2 near the lower-left corner to *r´* = *r* (1 + *U*) or *r´* = *r* (1 - *U*), respectively, where *U* is a constant characterizing the strength of the disorder, as depicted in Fig. S9(a). The model is in the QTI phase, with *B* = 0.3 T and *d* / *a* = 0.38 (Q1). As the disorder strength is increased, the eigenfrequencies of in-gap modes near the corner (represented as tan squares) experience slight shifts after a threshold value *U* = 0.28, as shown in Fig. S9(b). Experimentally, we implement the critical samples at *U* = 0.28 and compare the measurement results with sample Q1. Note that the semi- and quarter-cylinders near the boundaries of the samples are replaced by full circular cylinders with preserved areas of cross sections to facilitate the experimental fabrication as previous experimental realizations. The results in Fig. S9(c) shows a steady corner transmission at 8.23 GHz. The robustness of our corner states is thus proved.


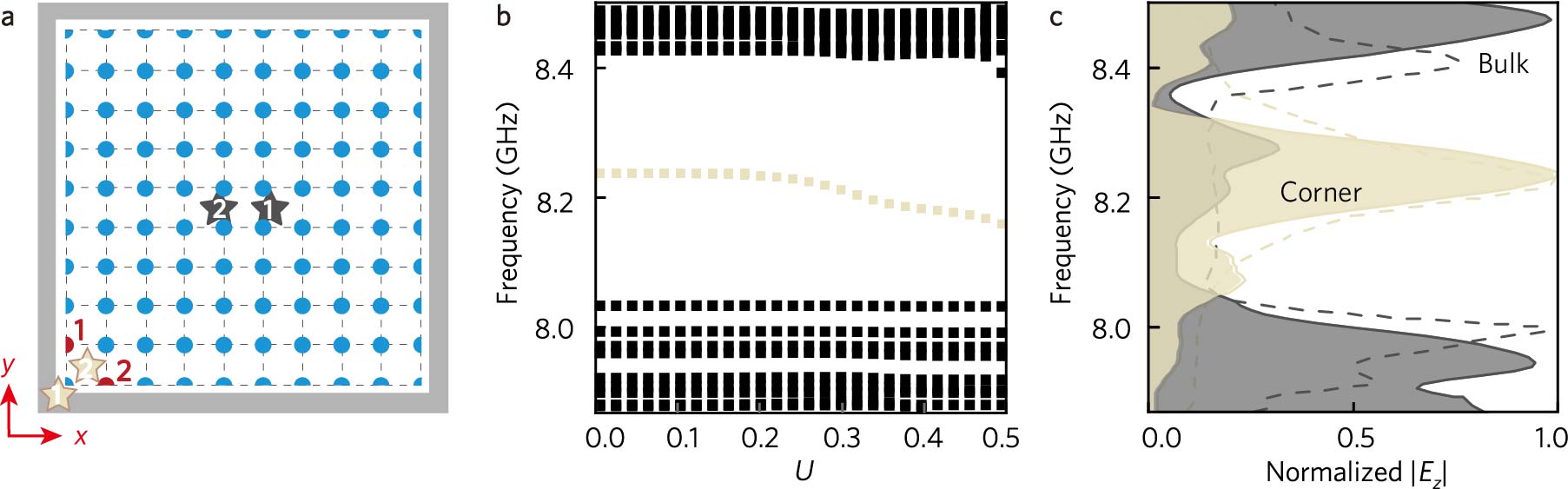


FIG. S9. Robustness of corner states. (a) Schematic of a quadrupole topological insulator with disorder. The radii of half rod 1 and half rod 2 (red color) are increased or decreased from *r* to *r´*= *r* (1 + *U*) or *r´*= *r* (1 - *U*), respectively. The black dotted lines frame square lattices. Colored stars denote the source antenna (labelled as “1”) and probe antenna (labelled as “2”) in microwave measurements for corner (tan color) and bulk (black color) transmissions. (b) Eigenfrequencies of sample in (a) as a function of disorder strength *U*. The tan squares are the corner states around the lower-left corner. (c) Measured frequency-resolved transmissions for *U* = 0.28 sample. The measurement setups are denoted in (a). The tan and black dotted lines show the results without disorder, i.e. *U* = 0.

For the sake of comparison, we also construct a trivial insulator with a defect near the corner, as illustrated in Fig. S10. Here, we follow the theoretical predictions [Ref. 25 in the main text] that the shift of square lattice atoms transfers a QTI phase into a trivial insulator phase. Hence, by moving the gyromagnetic cylinders of the sample in Fig. S9(a), this trivial insulator phase is achieved (see Figs. S10(a-b)). Subsequently, we introduce the defect by replacing a gyromagnetic cylinder near the lower-left corner with a dielectric cylinder having a permittivity of 2.2 (referred to as cylinder 3 in Fig. S10(c)). An in-gap defect state emerges in the trivial gap (Fig. S10(d) at *U* = 0). We then introduce additional disorder following a similar method used in Fig. S9(a). Figure S10(d) clearly demonstrates that the defect state is highly sensitive to disorder, leading to significant changes in its eigenstates as the disorder strength increases. A comparison between Fig. S9(b) and Fig. S10(d) shows that the observed corner state is indeed a topological quadrupole corner state, rather than being a defect state.


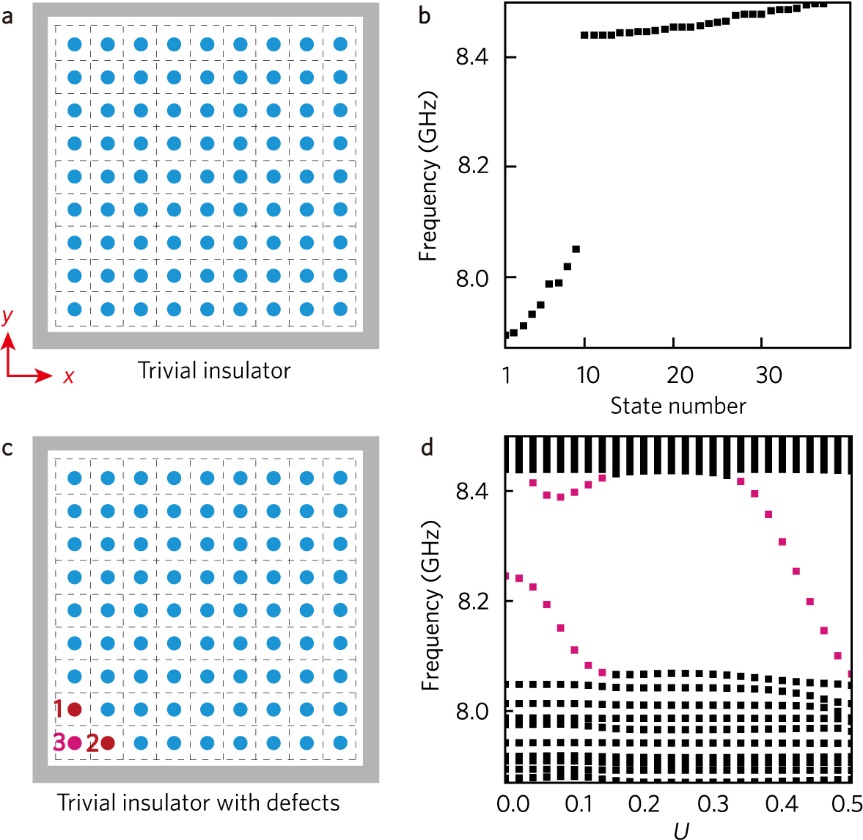


FIG. S10. Perturbation of defect states with disorders. (a) Schematic of a trivial insulator. The black dotted lines frame square lattices. (b) Eigenfrequencies of sample in (a). (c) Schematic of the trivial insulator with defect and disorder. Cylinders 1 and 2 are perturbed as the same way in Fig. S9(a). Cylinder 3 is a dielectric cylinder with permittivity of 2.2. (d) Eigenfrequencies of sample in (c) as a function of disorder strength *U*. The pink squares are the defect states.

**6. *Q* factor for corner states**

The topological protection in our gyromagnetic photonic crystal is attributed to the simultaneous presence of crystalline symmetries and broken time-reversal symmetry, which quantizes the quadrupole moment. However, such symmetries cannot protect the corner states from hybridizing with the same energy bulk states. Therefore, we explore the hybridization of localized corner states and bulk states numerically in an ideal QTI model, as manifested in Fig. S11. Here, the PhC sample is composed of two regions: the bulk region with lossy elements and the corner region with lossless elements. Loss is induced by complex dielectric constant *ε* = *ε'* + *iε"* with *ε'* = 14.3 and *ε"* = 1. As illustrated in Fig.4 in the main text, the corner states shift from the upper band continuum to the gap and then to the lower band continuum when the magnetic field is tuning from *B* = 0.18 T to 0.3 T and to 0.42 T. During the process, *Q* factor defined as *Q* = Re(*f*_0_)/[2Im(*f*_0_)] with *f*_0_ the complex eigenvalue of eigenstates, is given for eigenmodes that appear around the corner states (also including the corner states) in Fig.S11b. This parameter of corner states reaches a maximum for in-gap case (*B* = 0.3 T) and drops obviously both leftwards and rightwards due to hybridization with the lossy bulk modes. However, these corner states are still localized as shown by our experiments and simulations in the main text, and constitute the most general quadrupole topological features of corner-filling anomaly and fractional charge.


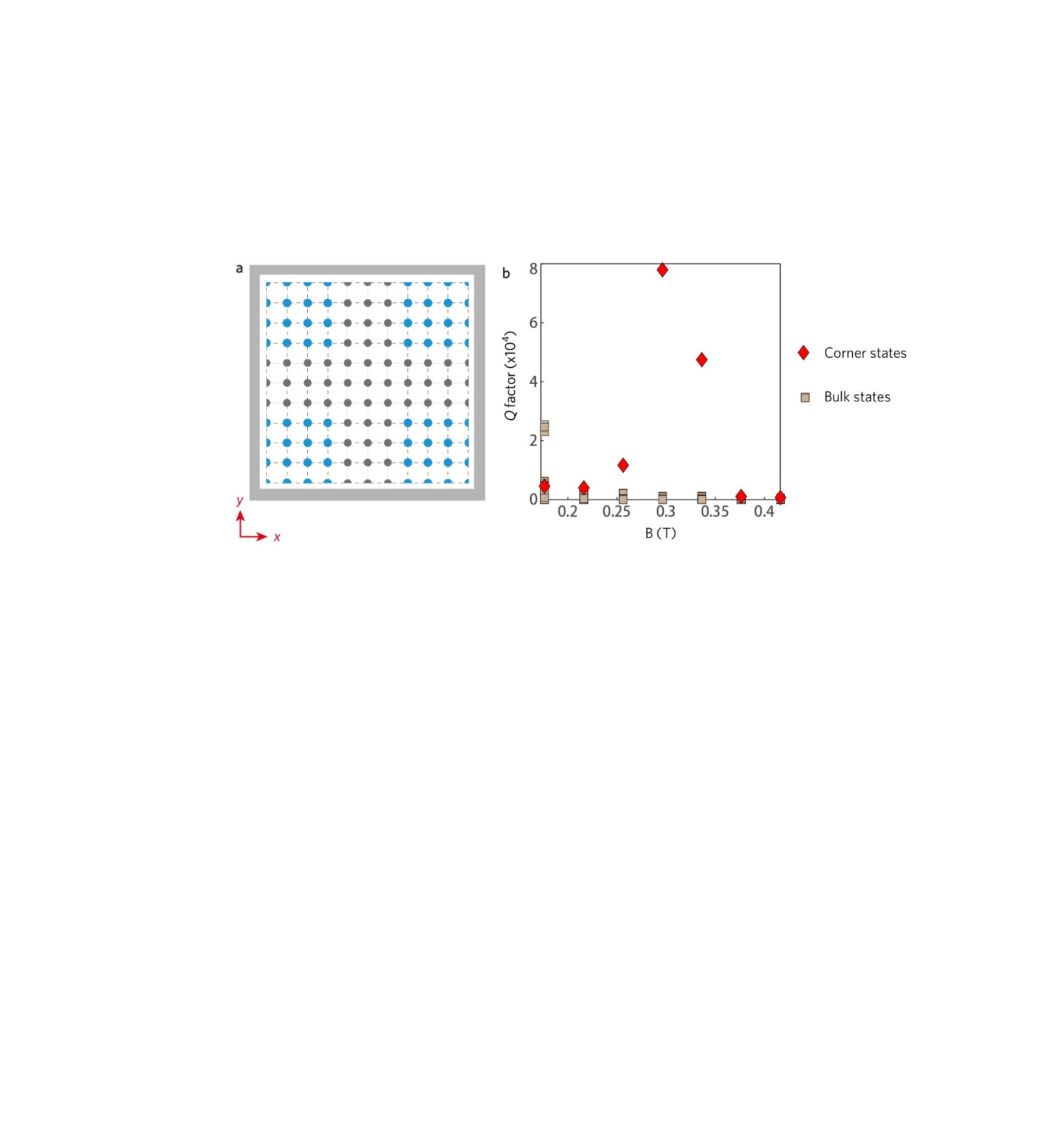


FIG. S11. Simulated *Q* factor for corner modes. (a) Schematic of the PhC sample. The blue dots indicate YIG rods without loss, while the dark gray dots represent YIG rods with dielectric loss. The black dotted lines frame square lattices. The gray region are PEC edges. This sample has geometrical parameters same to Q1 in the main text. (b) *Q* factor of eigen modes obtained at different magnetic field *B*. Here, ~ 20 eigen modes are calculated around the corner states’ frequency.

**References**

1. Zhang X, Lin ZK and Wang HX *et al*. Symmetry-protected hierarchy of anomalous multipole topological band gaps in nonsymmorphic metacrystals. *Nat Commun* 2019; **11**: 65.

2. Liu GG, Zhou P and Yang Y*et al*. Observation of an unpaired photonic Dirac point. *Nat Commun* 2020; **11**: 1873.

3. Zhou P, Liu GG and Ren X *et al*. Photonic amorphous topological insulator. *Light Sci Appl* 2020; **9**: 133.
